# Microbial-Derived Polyhydroxyalkanoate-Based Scaffolds for Bone Tissue Engineering: Biosynthesis, Properties, and Perspectives

**DOI:** 10.3389/fbioe.2021.763031

**Published:** 2021-12-21

**Authors:** Jian Li, Xu Zhang, Anjaneyulu Udduttula, Zhi Shan Fan, Jian Hai Chen, Antonia RuJia Sun, Peng Zhang

**Affiliations:** ^1^ Shenzhen Engineering Research Center for Medical Bioactive Materials, Center for Translational Medicine Research and Development, Shenzhen Institutes of Advanced Technology, Chinese Academy of Sciences, Shenzhen, China; ^2^ University of Chinese Academy of Sciences, Beijing, China; ^3^ Key Laboratory of Industrial Biocatalysis, Ministry of Education, Tsinghua University, Beijing, China; ^4^ Department of Chemical Engineering, Tsinghua University, Beijing, China

**Keywords:** polyhydroxyalkanoates, biopolyester, biodegradability, biocompatibility, synthetic biology, 3D rapid prototyping, bone tissue engineering

## Abstract

Polyhydroxyalkanoates (PHAs) are a class of structurally diverse natural biopolyesters, synthesized by various microbes under unbalanced culture conditions. PHAs as biomedical materials have been fabricated in various forms to apply to tissue engineering for the past years due to their excellent biodegradability, inherent biocompatibility, modifiable mechanical properties, and thermo-processability. However, there remain some bottlenecks in terms of PHA production on a large scale, the purification process, mechanical properties, and biodegradability of PHA, which need to be further resolved. Therefore, scientists are making great efforts *via* synthetic biology and metabolic engineering tools to improve the properties and the product yields of PHA at a lower cost for the development of various PHA-based scaffold fabrication technologies to widen biomedical applications, especially in bone tissue engineering. This review aims to outline the biosynthesis, structures, properties, and the bone tissue engineering applications of PHA scaffolds with different manufacturing technologies. The latest advances will provide an insight into future outlooks in PHA-based scaffolds for bone tissue engineering.

## Introduction

Bone tissue exhibits the unique ability of constant healing and remodeling during the lifetime of an individual (Stevens, 2008). It not only provides structural support ensuring adequate load-bearing capacity for the protection of the delicate internal organs of the body but acts as a mineral reservoir for the body to regulate the concentration of key electrolytes in the blood. It is generally believed that the bone has the capacity for spontaneous regeneration for relatively small defects; however, large bone defects, caused by traumatic injury, osteonecrosis, or osseous congenital deformities, would require surgical operation for restoration. The reconstruction of large bone defects remains a great challenge for researchers and orthopedic surgeons. Traditional therapeutic approaches of bony deficits are available, namely, using autografts, allografts, and xenografts (Fillingham and Jacobs, 2016; Raghuram et al., 2019). Autografts represent the current golden standard of bone transplantation, which possess the optimal characteristics of osteoconduction, osteoinduction, and osteointegration (Cypher and Grossman, 1996). However, their use is not without drawbacks such as supply limitation, variable resorption, and risk of donor site infection (Arrington et al., 1996; Nodarian et al., 2010). Furthermore, autografts often have limited ability to accelerate normal morphogenic and cellular events of fracture healing and remodeling (Mastrogiacomo et al., 2005). Alternatively, in some particularly important situations, when there is a large segmental bone defect, structural support is required, or when the capacity of the autograft is insufficient, allografts and xenografts can be used (Baldwin et al., 2019). A variety of problems associated with allografts and xenografts can be pathogen transmission, limited biological and mechanical properties, and rejection by the recipient’s body (Gruskin et al., 2012; Shibuya and Jupiter, 2015). To circumvent the deficiencies associated as reported, the most promising approach to solve the defects of bone transplantation is to study tissue engineering and related technologies to promote bone regeneration.

Bone tissue engineering (BTE) generally involves the employment of favorable biocompatible materials, combining with cells and bioactive factors, as engineered scaffolds that provide a specific microenvironment and architecture to support and promote tissue formations *in vivo* (O'Keefe and Mao, 2011; Peric Kacarevic et al., 2020). A great number of materials including natural materials or synthetic polyesters have been investigated as possible tissue-engineered scaffolds for bone and cartilage defect repair in recent decades (Agarwal and Garcia, 2015). Currently, natural biopolymers include chitosan (CS), collagen (Col), silk fibroin (SF), and synthetic polyesters such as poly(lactic-*co*-glycolic) acid (PLGA), poly(lactic acid) (PLA), polyethylene glycol (PEG), and polyurethane (PU), which have been widely used as a scaffold matrix for bone regeneration (Soundarya et al., 2018; Bharadwaz and Jayasuriya, 2020). An ideal scaffold suitable for BTE applications should not only provide initial mechanical strength and support but should also be degraded gradually and replaced by newly formed host tissue.

Polyhydroxyalkanoates (PHAs) are a class of structurally diverse natural polyesters, synthesized by various microbes and accumulated as intracellular storage compounds of carbon and energy in response to unbalanced culture conditions (Steinbuchel and Valentin, 1995; Chen, 2009). Owing to their exceptional biodegradability, inherent biocompatibility, modifiable mechanical properties, and thermo-processability, recently, PHAs as biopolymeric biomaterials have been widely exploited to design *in vivo* implants for potential therapeutic applications, such as cardiac tissue engineering (Shijun et al., 2016; Bagdadi et al., 2018), vascular tissue engineering (S. T. Cheng, Chen, and Chen, 2008; Zonari et al., 2012), nerve conduit tissue engineering (Hinuber et al., 2014; Schaakxs et al., 2017), and drug delivery vehicles (Pramual et al., 2016; Elmowafy et al., 2019). Very importantly, there is no evidence that PHAs or their biodegradation products are carcinogenic *in vitro* or *in vivo* (Peng et al., 2011; Raza et al., 2018a). However, there remain some bottlenecks in terms of PHA production on a large scale, purity, mechanical properties, and biodegradability, which need to be further addressed to make PHAs a realistic biomaterial. Therefore, scientists are making great efforts *via* synthetic biology and metabolic engineering tools to increase the product yields of PHAs at lower cost and develop PHAs with better physiochemical characteristics to widen biomedical application ranges.

We have divided this review into three parts. In the first part, we describe the background of PHAs including material properties, function of degradation products, teratogenicity and carcinogenicity of PHAs, and recent progress in synthetic biology and metabolic engineering on PHAs. In the second part, we summarize the manufacturing technologies for scaffold fabrication including conventional technologies and rapid prototyping. Finally, in the third part, an in-depth discussion is conducted with PHAs and PHA-based scaffolds for BTE, following the outlooks of future research emphasis.

## The Background of PHAs

### Material Properties of PHAs

PHAs, a similar lipid-like inclusion body, were first discovered in *Bacillus megaterium* and identified as the simplest member of PHAs called poly-3-hydroxybutyrate (PHB) (Lenz and Marchessault, 2005). After that, many different types of PHAs were found and served as storage materials of carbon sources and energy in response to extreme cellular conditions (Choi et al., 2020a). With the developed understanding of PHA biosynthesis, the first commercial production of PHAs was developed by Imperial Chemical Industries (ICI), who prepared PHAs into bioplastics which could replace petroleum-based plastics because of the oil crisis during the 1970s and because they have physical properties similar to those of polypropylene (PP) (Choi et al., 2020b).

**FIGURE 1:**
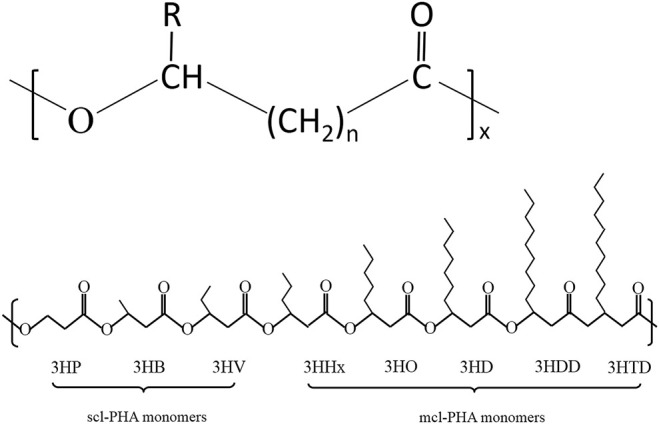
Structure and classification of PHAs. **(A)** General molecular structure of PHAs. n-range of variation from 1 to 4, x--range of variation from 100 to 35,000, R-alkyl group (C_m_H_2m+1_) or functionalized group; **(B)** Schematic diagram of structures of PHA monomers used to synthesize short-chain-length PHA and middle-chain-length PHA.

To date, more than 150 different PHA monomers have been reported. Depending upon the number of carbon atoms in the PHA monomer, there are three types: short chain length (scl-PHAs), medium chain length (mcl-PHAs), and long chain length (lcl-PHAs) (Li Z. et al., 2016; Lim et al., 2017). Figure 1 shows the general molecular structure of PHAs with their classification. As a class of polymers, PHAs not only have similarities in their physiochemical characteristics with conventional petroleum-based polymers like PP but also possess unique properties compared with other biopolyesters. A variety of PHAs offers a wide range of performance from hard crystalline to elastic. Table 1 summarizes the performance comparison of different PHAs with petroleum-derived polymers.

**TABLE 1:** Comparative overview of the physical properties of PHAs with conventional petroleum-based polymers.

Polymer		Melting temperature (°C)	Glass transition temperature (°C)	Elastic modulus (GPa)		Elongation to break (%)	Tensile strength (MPa)	References
Composition	Mol%									
Homopolymers								
PHB	100	171–180	4–10		3.5–4	0.4–5	11–40	Akaraonye et al. (2010); Ashby et al. (2002); Castilho et al. (2009); Sudesh et al. (2000)
P4HB	100	50–60	−48 to −51		149	1,000	104	Utsunomia et al. (2020); Jiang et al. (2012)
PHO	100	61	-		-	-	6–10	
Copolymers								
P(3HB-*co*-3HV)								
3HB:3HV	91–97:3–10	162–170	−4 to 2.2		-	-	19–38	Zhang et al. (2009)
	80–90:10–20	137–156	−1 to 1.7		0.8–1.2	50–100	20–32	Bengtsson et al. (2010); Zakaria et al. (2010)
	70–80:20–30	138–139	−6 to −0.1		1.37	30	70	Chanprateep et al. (2010); Ciesielski et al. (2015)
P(3HB-*co*-4HB)								
3HB:4HB	95:5	169	−2		1.23	10.7	1.36	Anjum et al. (2016); Chanprateep et al. (2010)
	90:10	159	-		-	-	24	Anjum et al. (2016); Doi et al. (1990)
	84:16	152	−8		-	-	26	Doi et al. (1990)
	76:24	161	−5		0.79	22.2	14	Chanprateep et al. (2008)
	36:64	50	−35		30	-	17	Chanprateep et al. (2008)
	10:90	50	−42		100	-	65	Chanprateep et al. (2008)
P(3HB-*co*-3HA)								
3HB:3HA	98:2	150–167	1		0.95	16	26	Chen et al. (2006)
	94–96:4–6	133	−8		0.2	680	17	Anjum et al. (2016); Hokamura et al. (2015)
P(3HB-*co*-3HP)	79–87:13–21	119.8–162.8	−3.1 to −2.1		-	-	-	Yu et al. (2007)
P(3HB-*co*-3HHx)	83–90:10–17	61–127	−1.2		0.5	113–400	9.4–21	Anjum et al. (2016); Zhang et al. (2009)
Terpolymers								
P(3HB-*co*-3HV-*co*-4HB)								
3HB:3HV:4HB	73:8:19	131	−10.0		0.10	316	12	Kumar et al. (2015)
	63:4:33	–	−14.0		0.10	937	9	
	49:18:33	–	−16.0		0.03	554	2	
	12:12:76	87.3	−21.1		0.14	9	4	Chanprateep and Kulpreecha (2006)
	11:23–24:55–56	92–100	−15 to −17		0.4–0.6	3–5	9–10	
	10:40:50	88	−13.7		0.12	300	9	
	4:3:93	55	−51.6		0.13	430	14	
P(3HB-*co*-3HV-*co*-3HHx)								
3HB:3HV:3HHx	75:13:12	101	−1.9		0.07–0.1	740–833	12.8–14.3	Bengtsson et al. (2010); Zhang et al. (2009)
	70:25:5	129	−7.2		–	–	–	
	67:20:13	58–68	−6 to −3.6		–	–	–	
	56:43:1	155	−5.5		–	–	–	
	48:24:28	54	−5.1		–	–	–	
	94:3:3	153–168	2.0		–	–	–	
High-density PE	100	112–132	–		0.4–1.0	12–700	18–33	Anjum et al. (2016); Castilho et al. (2009)
Low-density PE	100	88–100	−36		0.05–0.2	126–600	10–78	Anjum et al. (2016); Watanabe et al. (2009)
PP	100	170–176	−10		0.6–1.7	400–900	27–38	Singh et al. (2015)
Polystyrene	100	110–240	100		3.0–3.1	3–4	50	Capek (2005); Singh et al. (2015)
Polyvinylchloride	100	100–260	82		3.4	20–80	10–60	Singh et al. (2015)
PU	100	195	3400		0.004	-	38	Tian (2020)
Nylon-6,6	100	265	–		2.8	60	83	Anjum et al. (2016); Menezes et al. (2018); Singh et al. (2015)
Polypropylene-terephthalate	-	262	3,400		2.2	7,300	56	Anjum et al. (2016)

scl-PHAs are stiff and fragile due to their higher degree of crystallinity in the range of 55%–80% (Anjum et al., 2016; Mozejko-Ciesielska and Kiewisz, 2016). As a member of the scl-PHA family, the properties of PHB have been explained thoroughly because of its wide study. Compared with petroleum-based polymers such as PP, PHB has good thermoplastic properties, but its mechanical properties including Young’s modulus and tensile strength are poor. Moreover, PHB is optically pure and possesses piezoelectric material properties that contribute to the process of inducing osteogenesis (Philip et al., 2007; Bugnicourt et al., 2014). In contrast, another scl-PHA, poly-4-hydroxybutyrate (P4HB), possesses strong and malleable mechanical properties and has a similar tensile strength to polyethylene (PE). It has extremely elastic properties with elongation at break of approximately 1,000%, compared with PHB, which has an elongation at break of less than 10%. The material properties of P4HB can be changed when combined with other hydroxyl acids (Martin and Williams, 2003). Nevertheless, the high crystallinity, high melting point (*T*_*m*_), and low glass transition temperature (*T*_*g*_) make the scl-homopolymers relatively stiff and brittle and limit the range of applications. Therefore, the introduction of different secondary PHA monomers such as 3-hydroxypropionate (3HP) and 3-hydroxyvalerate (3HV) into the copolymers is an alternative strategy, and it greatly increases the flexibility and toughness of the polymer.

mcl-PHAs have properties that are great different from those of scl-PHAs. mcl-PHAs are flexible and behave as semi-crystalline elastomeric materials with low thermal temperature, low tensile strength, and higher elongation at break, for example, poly(3-hydroxyhexanoate-*co*-3-hydroxyoctanoate) (P3HHx3HO) (Rai et al., 2011; Reddy et al., 2003). mcl-PHAs serve as an elastomeric biomaterial under a certain side chain length. With the further increase of the side chain length, they become more sticky or viscous (Hazer and Steinbuchel, 2007). Furthermore, there are also scl–mcl-copolymers composed of scl- and mcl-monomers, for example, PHBHHx, which is suitable for different commercial/biomedical applications because it combines the thermomechanical properties of PE with the physiochemical properties of polyesters. Also, compared with the ductility and process ability of copolymers, those of PHA terpolymers are significantly improved. The PHA terpolymer poly(3-hydroxybutyrate-*co*-3-hydroxyvalerate-*co*-3-hydroxyhexanoate) (PHBVHHx) was synthesized and contained 39 mol% 3HV and 3 mol% 3HHx, whose tensile strength was 12 MPa and elongation at break was 408% while having no melting point. PHAs can be easily soluble in chloroform and other chlorinated solvents.

### Biocompatibility and Biodegradability of PHAs

The biocompatibility of the material means that it does not have any adverse effect on a living organism. Good biocompatibility of biomaterials for tissue engineering is a prerequisite for transplantation in humans or animals. Various available PHA materials were tested to demonstrate their biocompatibility both *in vitro* and *in vivo*. It is easy to prove the biocompatibility of PHB and P4HB because their degradable products, 3HB and 4HB, are natural metabolites in the human body. 3HB exists in concentrations of 0.03–0.1 mg/ml in human blood, while 4HB is widely distributed in the brain, lung, heart, liver, muscle, and kidney and excreted as carbon dioxide (Nelson et al., 1981). Moreover, low-molecular-weight PHB complexed with low-density protein and albumin has also been found in blood serum. Cell cycle regulations are important for cell survival. A study by Ahmed et al. (2010) indicated that PHB was good for nerve regeneration, while PHBV was in favor of BTE. Furthermore, another type of PHA known as PHBVHHx has been discovered and tested with the human keratinocyte cell line HaCaT and human umbilical cord Wharton’s jelly-derived mesenchymal stem cells (WJ-MSCs) (Ji et al., 2009; Ji et al., 2008). It was found that cell adhesion and proliferation on PHBVHHx membranes were more significant than other tested materials. In addition, the PHBVHHx film also supported the osteogenesis of MSCs (Hu et al., 2009).

Biopolymers as biomaterials should come with the ability to disintegrate themselves into nonhazardous products to prevent inflammatory responses *in vivo*, which is another important aspect of tissue engineering. Hence, information regarding the biodegradation of PHAs is momentous for its exploitation as a biomaterial. The family of PHAs could be decomposed by many enzymes such as lipases and hydrolytic enzymes into their monomers or oligomers. Chenet et al. (2017) found that PHB scaffolds implanted subcutaneously in mice were resorbed 8 weeks after implantation. Simultaneously, osteoid tissue and blood vessel ingrowth into the scaffold was observed. Moreover, through subcutaneous implantation in rabbits, the *in vivo* tissue reaction and biodegradation of PHBHHx, PLA, PHB, PHBHHx, and PEG blends of 1:1 and 1:5 were evaluated. It was shown that the degradation of PLA was the fastest, followed by PHBHHx and finally PHB (Qu et al., 2006). The results show that PHB has the slowest degradation rate because of its higher crystallinity. Thus, the intrinsic biodegradable properties of PHAs make them promising biomaterials in medical applications.

However, biodegradation of PHAs is not only affected by their properties, such as stereoregularity, crystallinity, chemical composition, and surface accessibility to PHA depolymerase, but also by a variety of factors including surface area, physical shape and form, pH, temperature, and moisture (Sudesh et al., 2000; Singh et al., 2019).

### Degradation Products of PHAs and Their Biological Functions

The cellular response of PHA degradation products including oligomers (oligo-hydroxyalkanoates, OHAs) and monomers (3-hydroxyalkanoic acids, HAs) is a key factor reflecting the biocompatibility of PHA for tissue engineering applications. The cell viability of mouse fibroblast L929 cells was not significantly affected by oligomer concentration without exceeding 20 mg/L, while the concentration of OHAs exceeds 40 mg/L, resulting in decreased cell viability, increased apoptosis, increased death, and prolonged cell cycle (Sun et al., 2007). 3-Hydroxybutyrate (3HB) is the main degradation product of PHB or its copolymers, namely, a ketone body, which is produced by the breakdown of long-chain fatty acids in the liver. 3HB has a short half-life and good tolerance to humans. Therefore, 3HB as a kind of nutrition or dietary compositions can be administered orally or intravenously to increase the level of ketone body in the blood to treat some diseases (Martin and Williams, 2003). A study showed that cell proliferation and DNA synthesis for several cell types were enhanced when treated with concentrations of 3HB ranging from 5 to 100 mg/L (Cheng et al., 2005). Furthermore, 3HB had an obviously stimulatory effect on cell cycle progression mediated by improving the intracellular Ca^2+^ concentration (Cheng et al., 2006). Table 2 summarizes the degradation products of PHAs and their applications both *in vitro* and *in vivo*.

**TABLE 2:** Degradation products of PHAs and their applications.

Degradation products of PHAs	Functions and applications	References
OHB, O3HB4HB, OHBHHx, OmclHA	OHAs in concentrations lower than 20 mg/L did not significantly affect L929 cell viability, while OHAs over 40 mg/L reduced cell viability with more apoptosis, more cell death, delayed cell cycle, and reduced cell proliferation. The cytotoxicity of OHAs decreased with increasing OHA side-chain length	Sun et al. (2007)
OHB, O3HB4HB, OHBHHx	The effect of OHBHHx was the best among all materials tested for gap junction intercellular communication of cells; OHBHHx was especially not harmful to the beta cells and could upregulate extracellular insulin secretion	Yang et al. (2002); Yang et al. (2002)
3-Hydroxybutyrate (3HB)	The cell proliferation and DNA synthesis of cell lines including murine fibroblast L929 cells, human umbilical vein endothelial cells (HUVECs), and rabbit articular cartilages (RACs) were enhanced when treated with concentrations of 3HB ranging from 5 to 100 mg/L. Furthermore, 3HB could obviously inhibit apoptosis and necrosis of L929 cells in high-density cultures	Cheng et al. (2006); Cheng et al. (2005)
3HB	ALP and calcium deposition, which are important biomarkers of mesenchymal stem cell osteogenic differentiation, were significantly intensified in direct proportion to the concentration of 3HB when it was lower than 10 mg/L. Besides, 3HB was an effective bone growth-stimulating agent and able to increase bone mass in mice under a microgravity environment	Cao et al. (2014); Zhao et al. (2007)
3HB	3HB could be utilized as an efficient enzyme-stabilizing and enzyme-protecting additive	O'Connor et al. (2013)
3HB	3HB could inhibit the NLRP3 inflammasome by preventing K+ efflux and reducing ASC oligomerization and speck formation to decrease interleukin IL-1β and IL-18 production in human monocytes	Youm et al. (2015)
3HB	3HB was found to attenuate atherosclerosis in mice by reducing the proportion of M1 macrophage and promote cholesterol efflux through its receptor G-protein-coupled receptor GPR109A	Zhang et al. (2021)
3HB	Increasing 3HB level attenuated NLRP3 inflammasome formation and antagonized proinflammatory cytokine-triggered mitochondrial dysfunction and fibrosis to ameliorate heart failure with preserved ejection fraction (HFpEF) pathogenesis	Deng et al. (2002)
3-Hydroxydecanoic acid (3HD)	Conjugation of anticancer DP18L peptide with 3HD derived from mcl-PHA enhances its anticancer activity; (R)-3-hydroxyalkanoic acids with 9 and 10 carbons were most effective at increasing DP18L activity	Szwej et al. (2015)
3-Hydroxyoctanoic acid (3HO)	The presence of the carboxylic group was found important for antimicrobial activity; 3HO derivatives were inhibitory against a variety of microorganisms but not to mammalian cells	Radivojevic et al. (2016)
3HB methyl ester (3HBME)	3HBME was able to inhibit cell apoptosis under glucose-free condition, rescued activities of mitochondrial respiratory chain complexes that were impaired in Alzheimer’s disease patients, and decreased the generation of ROS	Zhang et al. (2013)
3HBME	3HBME could be used as an effective neural protective agent to obviously decrease the apoptosis ratio of mouse glial cells by signaling pathways related to the elevation of cytosolic Ca^2+^ concentration. Mice treated with HBME performed distinctly better in the Morris water maze than either the negative controls or positive controls. It indicated that 3HBME improved learning and memory of mice	Obruca et al. (2016); Xiao et al. (2007)
3HBME	3HBME attenuated the development of osteoporosis in mice under a microgravity environment, helping prevent deterioration of bone microstructure and mechanical property	Cao et al. (2014)

The effect of 3HB as an osteopromotive factor on bone formation was demonstrated. The pivotal biomarkers including alkaline phosphatase (ALP) activity and calcium nodule deposition of osteogenesis of murine pre-osteoblast MC3T3-E1 were remarkably intensified, correlating linearly with the concentration of 3HB when it was no more than 10 mg/L. *In vivo*, serum calcium deposition and ALP activity were significantly increased when the 3-month-old ovariectomized rats were treated with 3HB for 12 weeks; osteocalcin was also reduced. Simultaneously, the femur biomechanics was obviously enhanced due to the improved bone mineral density and trabecular bone volume (Zhao et al., 2007). On top of that, the effect of 3HB and its derivative 3-hydroxybutyrate methyl ester (3HBME) on mice was investigated under a simulated microgravity environment (Cao et al., 2014). It was found that bone loss was quickly alleviated when mice were treated with 3HB or 3HBME. This was due to the nuclear factor of activated T-cell cytoplasmic 1 (NFATc1) of pre-osteoclast differentiation being downregulated by 3HB or 3HBME. Decreased transcriptional activity of NFATc1 reduced the number of osteoclasts; thus, the bone tissue absorption was effectively inhibited to ameliorate bone loss in microgravity. Recently, new evidence suggested that 3HB could inhibit the NLRP3 inflammasome rather than undergoing oxidation in the mitochondria to reduce the production of interleukin IL-1β and IL-18 in human monocytes (Youm et al., 2015). Intriguingly, 3HB was also used to decrease the proportion of M1 macrophage and accelerate cholesterol efflux by acting on macrophages through its G-protein-coupled receptor GPR109A to attenuate atherosclerosis in mice (Zhang et al., 2021).

Additionally, the degradation product of polymer P4HB contains P4HB oligomers and 4HB monomers, which can be adopted as drugs to treat some mental diseases such as neurosis, chronic schizophrenia, and Parkinson’s disease (Maitre et al., 2016; Utsunomia et al., 2020). So far, there is no evidence that has been reported regarding any cytotoxicity caused by degradation products of PHAs.

### Non-teratogenicity and Non-carcinogenicity of PHAs

Many investigations indicated that PHAs have been used as drug delivery vehicles and grafts to support tissue regeneration. However, the possibility of teratogenicity and carcinogenicity induced by PHA matrices is an issue of concern. So far, only little information reports are available with respect to the implication of biodegradable PHAs on the growth of embryos as well as larvae. Li et al. (2016a) carried out a significant experiment to evaluate the influence of PHAs on growth profile of zebrafish larvae. It was found that there were no adverse negative effects of PHA polymers on the growth and development of zebrafish embryos as well as larvae.

Furthermore, Peng et al. (2011) performed interesting research to demonstrate the effect of fast-growing cells on PHA matrices toward tumor progress. The results indicated that no carcinogenic signal was found in least eight passages when the proliferation of rat osteoblasts was cultivated on the films of various PHAs. This observation further demonstrated the normal condition of cells growing and developing on the PHA polymers. This study revealed that common PHAs might be exploited as safe implant materials for supporting cell growth without a sign of vulnerability toward tumor formation.

From the above studies, available PHAs might be utilized as safe implant materials in different medical applications to support cell and tissue growth with very limited negative effects.

### Synthetic Biology and Metabolic Engineering Strategies for PHA Production

**FIGURE 2:**
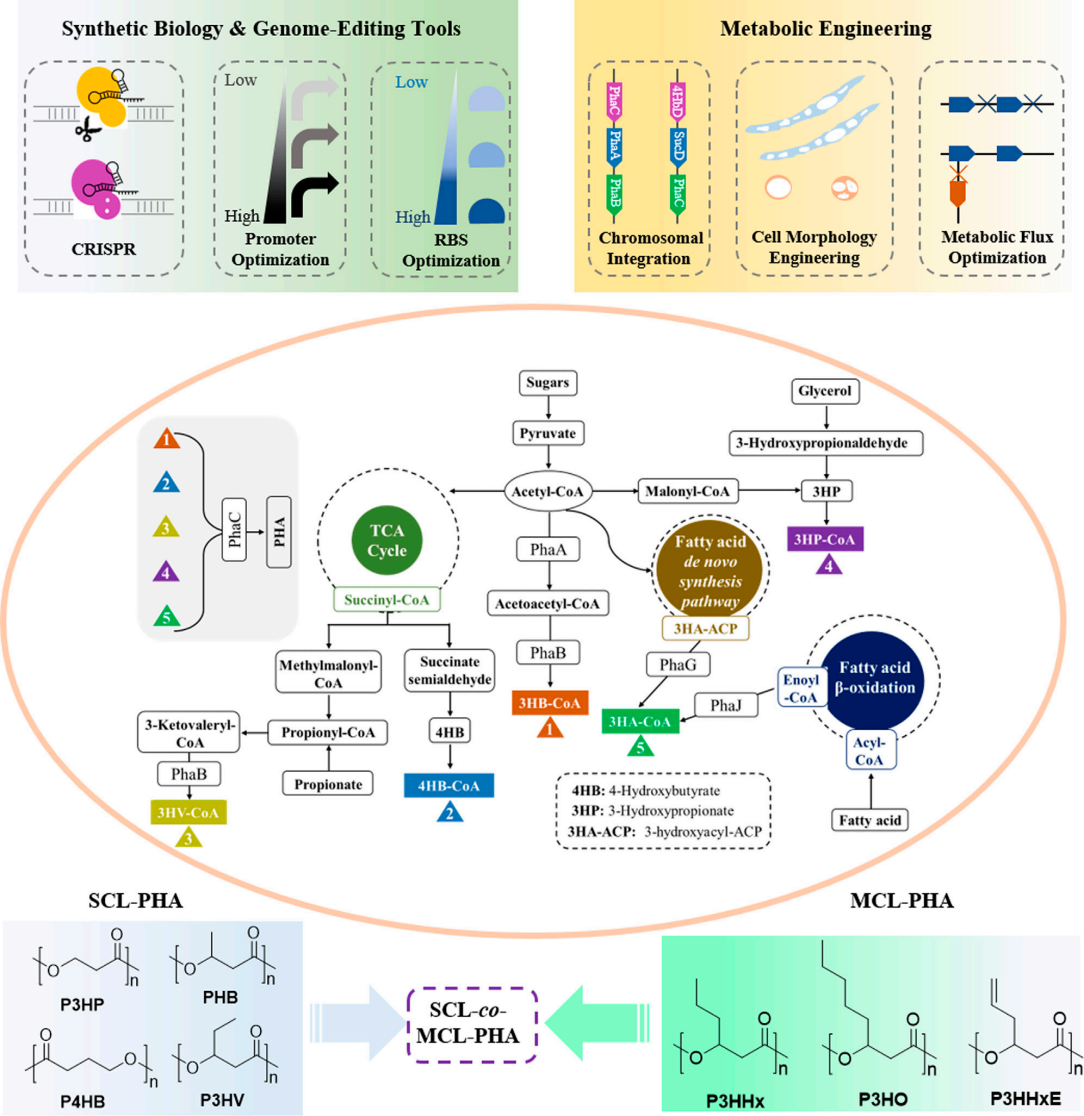
Metabolic pathways of PHA with synthetic biology and metabolic engineering strategies for production of PHA.

Although PHAs are potential candidates for biomedical materials, the high cost of manufacturing PHAs is the main constraint that limits its range of applications on an industrial scale. Therefore, enhanced PHA production strategies based on synthetic biology and metabolic engineering are being developed and utilized to lower the production cost of PHAs (Figure 2).

Synthetic biology as a tool involving mathematics and systems biology has been utilized to design biological parts and biosystems with new or improved properties, whereas metabolic engineering focuses on the engineering of microbial cell factories for production of fuels and chemicals using DNA-editing approaches (Keasling, 2014; Keasling, 2008; Schwille, 2013). Synthetic biology tools have increasingly been employed to solve scientific and technical challenges in metabolic engineering. In recent years, there have been incremental studies on account of synthetic biology and metabolic engineering technologies to improve the PHA product yields at lower cost and their development in a broad range of microorganisms including *Halomonas bluephagenesis* (Chen et al., 2016; Meng and Chen, 2018; Zheng et al., 2020), *Ralstonia eutropha* (Raberg et al., 2018; Bhatia et al., 2019), *Pseudomonas putida* (Prieto et al., 2016; Weimer et al., 2020), *Aeromonas hydrophila* (Mozejko-Ciesielska et al., 2019), *Haloferax mediterranei* (Lu et al., 2008; Lin et al., 2021), and recombinant *Escherichia coli* (Rahman et al., 2013; Jung et al., 2019).

More recently, Tan et al. (2011) reported a halophilic chassis strain named *Halomonas bluephagenesis* TD01 (*Halomonas bluephagenesis* TD01), which was successfully exploited for PHA production under non-sterile and continuous fermentation conditions for 14 days (Tan et al., 2011). Moreover, in *H. bluephagenesis* TD01, genetic manipulation was implemented by metabolic engineering or synthetic biology to facilitate different PHA fabrication or production. For example, using low-cost glucose as single carbon source to synthesize P34HB with a high ratio of 4HB, the recombinant *H. bluephagenesis* TD01 harboring two interrelated 4HB biosynthesis pathways including four heterologous genes encoding 2-oxoglutarate decarboxylase (ogdA), succinate semialdehyde dehydrogenase (gabD), 4-hydroxybutyrate dehydrogenase (4hbD) and 4-hydroxybutyrate-CoA transferase (orfZ), endogenousgene controlled expression of succinate semialdehyde dehydrogenase (gabD) was knockout, which was competed with 4HB synthesis flux, increasing the molar ratio 4HB from 0.17 to 17.04 mol% (Ye et al., 2018). On the other hand, CRISPR/Cas9 gene-editing system was constructed in *H. bluephagenesis* TD01, resulting in achieving the deletion and insertion of 4.5 kb gene, which only took about 3 weeks in *H. bluephagenesis* TD01 (Qin et al., 2018). Using this gene-editing system, the *prpC* gene, encoding 2-methylcitrate synthase, in *H. bluephagenesis* TD01 was deleted for enhanced capacity of the *ΔprpC* mutant to synthesize PHBV with increasing 3HV fraction approximately 16-folds (Qin et al., 2018). The convenient and scaled genome-editing approach vastly quickens up the development of *H. bluephagenesis* TD01 as chassis of the next generation industrial biotechnology (NGIB) (Chen and Jiang, 2018).

Recent years, recent advances in synthetic biology including CRISPR gene-editing system, promoter, and ribosome-binding site (RBS) optimization, and metabolic engineering containing chromosomal integration, cell morphology engineering, and metabolic flux optimization, have been employed to manipulate microorganisms to gain more efficient PHA production at low cost. Figure 2 shows the major biosynthesis pathways of PHA with possible engineering strategies for production of PHA (Choi et al., 2020b; Zhang et al., 2020). In addition, with the discovery of more novel and effective synthetic techniques, various PHAs with excellent performances including homopolymers, copolymers, block polymers, and functional polymers will be designed and produced. These will promote PHA materials in the application of regenerative medicine on its way.

## Manufacturing Technologies for Scaffold Fabrication in Tissue Engineering

Currently, various methods have been utilized to fabricate PHA scaffolds. In this section, the conventional techniques to create PHA scaffolds will be discussed including electrospinning, particulate leaching, thermally induced-phase separation (TIPS) and solvent casting methods. In addition, this section discussed the 3D printing technology designed functional PHA scaffold systems.

### Conventional Techniques for Scaffold Fabrication

The simple and effective strategies of solvent casting and particulate leaching have been used to fabricate scaffolds. These methods are intended to develop polymeric solutions with uniform salt particles distribution and certain size by dissolution in specific organic solvents. Followed by the formation of 2D films or 3D scaffolds by evaporation of solvents to lead to the reinforcement of slat particles into the matrix system. Further, they were immersed in ddH_2_O to remove any unreacted salt by leaching process to generate the extremely porous network. The porosity, shape, and size could be well controlled by the usage of the porogen (Plikk et al., 2009; Hokmabad et al., 2017). The composites of PHBV- β-Ca_2_SiO_4_ were fabricated using solvent cast and particle leach methods by Wang et al. They studied the porosity and osteogenic properties of composites and confirmed the 87% porosity and good cell attachment, the proliferation of MG-63 cells respectively. Also, they mentioned that usages of organic solvents in this method are a major disadvantage due to their toxic nature to cells, bioactive small molecules and proteins (Wang et al., 2013).

Thermally induced phase separation (TIPS) for the fabrication of scaffolds is commonly used to prepare 3D scaffolds. This method involves quenching of the polymer solution and undergoing a liquid-liquid phase separation including rich and poor phases of polymers. Subsequently, the rich and poor phases of polymers endure the solidification and sublimation process to form a scaffold with well interconnected porous. The mechanical properties, pore morphology and the porosity of the scaffold can be disturbed by the type of polymers/solvents and their concentrations and quenching and phase separation temperatures (Rowlands et al., 2007). Li et al. (2008) reported that maleated PHBHHx scaffolds were fabricated by TIPS. They compared the maleated PHBHHx with pure PHBHHx, the maleated PHBHHx scaffolds showed virtuous behavior in terms of surface energy/charge, hydrophilic and porosity, which encouraged the promising growth of cells.

Electrospinning is an attractive technique to produce fibrous scaffolds with required morphology, porosity and diameter distribution ranging from nano to submicrometer. Nanofibrous scaﬀolds exhibit superior biological properties than non-nanofibrous due to their high surface-to-volume ratio and well interconnected porous network which are easier for cell communications and therefore they are considered as potential candidates for tissue engineering applications (Xue et al., 2019). PHB and PHBV electrospun scaffolds were fabricated with a diameter of micro and nano size using the electrospinning method (Kundrat et al., 2019; Kaniuk et al., 2020; Kim et al., 2020). The electrospun fibers exhibited controlled surface hydrophobicity to the benefit of cell growth and infiltration (Volova et al., 2014). Interestingly, Wang et al. (2012) developed PHBHHx-based spun scaffolds and examined their biological behavior by exposing them to rat-derived MSCs and proved their differentiation into osteoblasts. Also, they fabricated the randomly oriented and well aligned PHBHHx-based spun fibers scaffolds. The regular alignment of fibers enhanced the proliferation and differentiation of MSCs when compared with randomly oriented fibers networks. It’s well-known that the electrospinning technique is highly skilled in reproducing fibrous scaffolds for tissue engineering applications. However, this technique has a major obstacle in controlling the porosity of scaffolds for a wide range of clinical applications.

### 3D Printing for PHA Scaffold Fabrication

3D printing technique works based on additive manufacturing principles. This technique is highly recommended for fabricating 3D scaffolds with controlled porous internal design, desired shape, and size (Mota et al., 2015). Also, this method could be adopted to incorporate biological agents and small biomolecules into scaffold systems while production of 3D scaffolds. 3D printing involves a layer-by-layer process that includes diﬀerent types of techniques such as direct ink writing (DIW), fused deposition modeling (FDM) and selective laser sintering (SLS). Among these techniques, the 3D printing method is an adequate technique to generate particular scaffold structures with appropriate microenvironments for better cellular functions including infiltration, cell adhesion, proliferation and differentiation. Table 3 shows the comparison of traditional and 3D printing techniques used to fabricate scaffolds.

**TABLE 3:** Comparative overview of manufacturing technologies for scaffold fabrication.

Techniques	Advantages	Disadvantages	References
Solvent-casting and particle leaching	Easiness and low cost	Use of organic solvents	Goh and Ooi (2008); Ye et al. (2018b)
		Limited to 2D structure	
		No customization	
Freeze-drying	Retained bioactivity	High time and energy utilization	Wu et al. (2010)
	Controllable pore size	Use of organic solvent	
Gas foaming	Good mechanical strength	High heating requirement	Ji et al. (2012)
	High porosity	Poor interconnection of pore structures	
Sol-gel technique	Formation of a variety of structures	Use of organic solvents	Figueira (2020)
		Weak mechanical strength	Zheng and Boccaccini (2017)
TIPS	Simple controlling process	Use of organic solvents	Li et al. (2008); Rowlands et al. (2007)
	Less affinity of defect formation	Time-consuming	
	Various microstructures of scaffolds	No control on final geometry	
Emulsification	High surface area-to-volume ratio	Limited design freedom	Piacentini et al. (2017)
		No significant 3D development	
Electrospinning	High surface area-to-volume ratio	Use of organic solvents	Xue et al. (2019)
	Controllable porous construction	Difficulties to achieve 3D structure	
DIW	Construct internal channels	Difficult to remove unbounded powder from curved channels	Li et al. (2019)
	Retained bioactive molecules		
FDM	Excellent mechanical strength	Generation of fibers with regular size and molten phase of material requirement	Vyavahare et al. (2020)
	Good porosity		
	No need of toxic solvents		
SLS	Good accuracy	High-cost running process	Mazzoli (2013)
	Short time process	Release of toxic gases	
	No need of post-process		
Stereolithography	Fabrication of complex internal structures	Limited to photopolymers	Melchels et al. (2010)
	Retained bioactive molecules		

DIW is an extrusion-based 3D printing technique (Li et al., 2019). In the fabrication of PHA scaffolds, the ink is generally prepared by dissolving the PHA particles into an organic solvent to form a homogeneous solution with rheological properties. The ink is extruded through a thin nozzle by a computer controlled robotic deposition system, as well as supporting its own weight during assembly. After printing, the cooling or drying need to be adopted to completely remove the organic solvent from the scaffold.

SLS is a solid freeform fabrication technique, in which various compositions of nanopowders are used to create physical models *via* a selective solidification process. Diermannet al. (2019) investigated the degradation behavior of high molecular weight PHBV scaffolds manufactured by SLS without using predesigned porous architecture. The results indicated that the manufactured PHBV scaffolds using SLS also exhibited adequate mechanical properties and satisfactory structural integrity even if immersed in phosphate-buffered saline solution for 6 weeks. Henceforth, they proved that PHBV has good thermal stability and could be a highly cost-effective choice for the fabrication of scaffolds. Duan et al. (2011), developed scaffolds with the combination of PHBV and calcium phosphate (CaP) by SLS for bone regeneration applications. They developed reproducible PHBV-CaP scaffolds by optimization of several factors of the SLS technique such as scan, speed, laser power and thickness of layers. Finally, they proved that SLS has the competency to produce good standard and refined complex architecture with porous structures to meet the tissue engineering criteria.

FDM is a layer-by-layer melt-extrusion approach that consists of heating the biopolymer above its glass transition temperature to form a thick solution and then deposing the extruded material still hot to render the adhesion with the underneath layers which already cooled down and hardened. Despite FDM being considered as the promising 3D printing technique in a wide range of applications, with different polymers, its utilization for PHA biomedical devices is still extremely challenging. Because of the narrow melt processing temperature window of this technique hinders its application in production of 3D PHA porous scaffolds. So far, only four scientific research works have been published, and they evaluated the applicability as preliminary investigations (Kosorn et al., 2017; Wu and Liao, 2017; Ausejo et al., 2018).

In conclusion, 3D rapid prototyping has been used for processing various micro-structures with controllable factors including shape/size and interconnectivity of pores, which was suitable for cell growth and rapid nutrient diﬀusion. However, the above-mentioned techniques have their pros and cons. Though, the adopted technique could be more focused on fulfilling the necessities of the particular tissues to be regenerated or healed.

## PHAs and PHA-Based Scaffolds for BTE

### Blending, Surface or Chemical Modification for Improved Application Properties of PHAs

PHAs including PHB, P4HB, PHBHHx, PHBV and PHO have been investigated materials for tissue regeneration (Ray and Kalia, 2017). However, PHAs are kinds of natural long chain fatty acid molecules with hydrophobic properties and lack of modifiable active groups. These features make them inconvenient for use in various applications, although PHAs show their excellent biodegradability. To enhance their usage, several strategies including blending, surface and chemical modifications have been employed to change their physiochemical characteristics to fulfill the requirements of medical applications. The blending option can be valuable considered for developing new kinds of polymers with enhanced performance, and the disadvantages of the parent polymers can be evitable. Therefore, PHA family members blending with each other or with other polymers can significantly improve PHA performance by choosing the proper compositions of the blend and preparation conditions, which is beneficial for expanded applications. Yang et al. (2002) systematically studied the blending of PHB with PHBHHx and their biomedical applications. The results uncovered the growth of L929 on the blending films exhibited marked improvement than growth on PHB films, which demonstrated that the blends have better biocompatibility contributed by PHBHHx. The following detailed studies have revealed that the surface of pure PHB film is the existence of many pores and protrusions, which are caused by PHB with a high degree of crystallization and rapid rate of crystallization. This rough morphology surface may be responsible for inhibiting cell adhesion and growth. However, the crystallization behavior of PHB decreased dramatically when PHBHHx was added to PHB. As a result, the films of PHB and PHBHHx blending exhibited the regular and smooth surface, which availed cell adhesion and growth. In another study, the physical properties of PHBHHx such as thermal stability, flexibility, and mechanical strength were improved when PHBHHx was blended with P34HB. The PHBHHx/P34HB blend exhibited the roughest surface and had the highest viability of chondrocytes when the weight ratio of PHBHHx/P34HB is 4:2. The results indicated that this blend could be beneficial to cartilage tissue engineering (Luo et al., 2009).

PLA is a linear biopolymer chemically synthesized from lactic acid that has been utilized as implant material with FDA approval. However, the extensive adoption of PLA at the commercial level has been hindered by the low-impact toughness and low end-use temperature. Hence, to overcome the drawbacks associated with PLA, fillers and impact modifiers are often added into PLA. Hence, blending PLA with PHA was considered as an effective method to change the properties of polymers. Several studies have focused onthe blend of PHA with PLA. Gerard and Budtova (2012) indicated that PLA/PHBV blends served a significant role in improving the thermal stability of PHBV. Even though PLA and PHBV are brittle polymers, blends containing a small amount of PHBV into PLA matrix showed an obvious ductile plastic deformation. Besides, various ratios of PHB and PEG were blended to form films used to test blood compatibility. The results found that PEG played an essential role in delaying clotting time and reducing thrombocyte adherence (Cheng et al., 2003).

In addition to blending, surface modification is another simple and effective approach for expanding the application of PHAs (Xue et al., 2018). Although PHBHHx displays good mechanical properties compared with PHB, it is very difficult to introduce active groups into the polyester carbon chain of PHBHHx, which leads to its applications restricted. However, a study revealed that the hydrophilicity of PHBHHx could be increased by strong alkaline treatment to further avail cell growth. This was due to the presence of more charged hydroxyl and carboxyl groups produced on the surfaces of the PHBHHx after the treatments. Through this kind of modification, the surface area and surface roughness of the material were increased, which is beneficial for cell survival and growth. Furthermore, the surface hydrolyzed PHBHHx films significantly presented fibronectin adsorption and enhanced MC3T3-E1 cell attachment and proliferation compared to unmodified films (Li et al., 2005). The surfaces of PHAs can bind several amphiphilic proteins like PHA granule binding protein (PhaP), PHA synthesis repressor protein (PhaR) and poly-β-hydroxybutyrate depolymerase (PhaZ) through strong hydrophobic interaction. Arginine-glycine-aspartate (RGD) is a tripeptide involved in cell adhesion, and generally regarded as an efficient way to modify biomaterials for guiding cell behavior for tissue regeneration. Previous studies showed PhaP-RGD fusion protein was used to coat the films of PLA, PHBV and PHBHHx, which led to increasing polymer surface hydrophilicity to facilitate cell adhesion, spreading, migration, and proliferation compared with their corresponding uncoated films (Dong et al., 2010). Xie conducted a study (Xie et al., 2013), using a fusion protein abbreviated as PhaP-IKVAV to coat the films of PHBVHHx, PHBHHx and PLA respectively, which distinctly promoted the rat neural stem cells (NSCs) adhesion, proliferation and neural differentiation compared to uncoated materials. Furthermore, it was found that human vascular smooth muscle cells (HVSMCs) cultured on films of PHAs coated with PhaR-KQAGDV oligopeptide, which showed better cell adhesion and growth than controls (Dong et al., 2012).

Additionally, different methods of chemical modifications including chlorination, carboxylation, epoxidation, and grafting have been carried out on PHA modification (Moore et al., 2005; Hazer and Steinbuchel, 2007). Acquired new physiochemistry characteristics like convertible glass transition and melting temperatures, increased hydrophilicity will be endowed into the polymers of PHAs *via* chemical modifications (Arslan et al., 2007; Raza et al., 2018b). Therefore, chemical modification is the simple and controllable way to alter the polymer structure with predictable properties, which are beneficial to further enlarge the space of their applications.

### Intrinsic PHAs and PHA Composites-Based Scaffolds for BTE

PHAs have been studied as a suitable substrate for tissue engineering on account of their excellent biodegradability, biocompatibility and thermo-processability. Among kinds of tissue regeneration applications, especially, in BTE, various types of PHAs and PHA composites-based scaffolds have been frequently adopted to support bone tissue growth both *in vitro* and *in vivo.* Rabbit bone morrow-derived mesenchymal stem cells (rBMSCs) were cultured on PHBHHx, PHB, and PLA 3D scaffolds respectively to assess their osteogenesis responses (Wang et al., 2004). It was found that the morphology of the cells altered from spindle fibrous into osteoblast-like cells with round cell shape. More specially, the cells grown on PHBHHx scaffolds presented specific markers of osteogenesis including high alkaline phosphatase activity, strong calcium nodule deposition than that on PHB and PLA scaffolds respectively. This contributed by the suitable roughness of PHBHHx surface better for osteogenic differentiation, implicating that PHBHHx was a promising biomaterial for bone tissue regeneration.

More recently, Chen lab reported that highly open porous microspheres (OPMs) made of PHBVHHx were fabricated by a modified method of gas-in-oil-in-water double emulsion, namely, PHA OPMs, which possess excellent connections from internal pores to outside surface as injectable carriers for repair complexly shaped tissue defects (Wei et al., 2018). PHA OPMs supported stronger osteogenic differentiation of human bone marrow derived mesenchymal stem cells (hMSCs) *in vitro* compared with traditional PHA microspheres with less porous hollow and PLA OPMs. ALP activity detected on/in PHA OPMs was significantly higher than both PHA HMs and PLA OPMs groups on days 4 and 7 during osteogenic induction. In addition, abundant new bone tissue was observed growing into PHA OPMs than other tested materials in an ectopic bone formation mouse model. PHA OPMs seem to be a potential medical scaffold which are not only beneficial for osteogenic differentiation of hMSCs *in vitro*, but also helps to repair the bone defect *in vivo*. One study on P34HB fiber scaffolds were prepared by electrospinning, which obviously upregulated osteogenic-related genes expression of mouse adipose-derived stem cells such as Runx2, OPN and OCN with induction. This finding indicated that fiber scaffolds of P34HB are potential candidates for BTE (Fu et al., 2014).

However, to further improve the mechanical properties and bioactivity, various PHA composites-based scaffolds including hydroxyapatite (HAp), bioactive glass (BG) and ceramics have been extensively studied for the repairment of bone defects. When HAp was incorporated into PHB to be fabricated scaffolds, not only the compressive elastic modulus and maximum stress of the PHB/HAp scaffolds were improved, but also enhanced osteoblast responses to the scaffolds (Ramier et al., 2014; Ding et al., 2016). PHBHHx composite scaffold with polylactide-grafted hydroxyapatite as compatibilizer has been systematically investigated including superficial roughness and wettability, mechanical strength, as well as *in vitro* bioactivity (Ma et al., 2017). The results demonstrated that the attachment and viability of hMSCs improved when cells seeded on PHBHHx based composite scaffold combined with surface-graft HAp compatibilizer. Moreover, the osteogenesis related genes including COL I, Runx2, OCN and OPN were up-regulated, thus made for osteogenic differentiation, indicating that this kind of scaffold might be a suitable matrix as bone substitutes.

Overall, therefore, PHAs and PHA composites-based scaffolds are potential candidates for BTE.

## Conclusion and Future Perspective

It is still a major clinical challenge for reconstruction of large bone defects. Autografts, allografts, and xenografts as traditional therapeutic approaches have been restricted because of their disadvantages. In recent decades, a variety of materials including natural materials or synthetic polyesters have been investigated as possible tissue-engineered scaffolds for bone and cartilage defects repaired. PHAs are biopolyesters produced by many microorganisms. Due to the excellent biodegradability, biocompatibility, and adjustable mechanical properties, PHAs allow being exploited as great attractive biopolymeric biomaterials over other polyesters. More importantly, because of its slow degradation rate (6 months–2 years) and high hydrolytic stability *in vivo*, PHAs are more suitable for long-term applications, especially in the area of bone tissue regeneration.

In this review, the effect of degradation products such as 3HB, 4HB were discussed, including the improvement of cell adhesion, proliferation, and treatment of neurodegenerative disorders. However, in practical applications, PHAs still have only a very limited market in both clinical and industrial materials compared with mature conventional petroleum-derived polymers. There are two main reasons: 1) the major drawback is the high production cost of PHAs. 2) Unexploited manufacturing methods for PHA scaffolds and strong hydrophobicity of PHAs. To meet these challenges, two strategies can be adopted: first, constructing a strain chassis of producing diverse PHAs at low cost with high PHA yields, using a simple fermentation process. To achieve this goal, recently, scientists are dying of great efforts to make *halophiles* as a candidate of a super chassis by synthetic biology and metabolic engineering approaches for producing PHAs (Chen X. et al., 2017). For example, Chen and co-workers indicated that diverse properties of PHAs with high cell density and high substrates conversion rate could be produced by *H. bluephagensis* TD01 (Ye H. et al., 2018; Ling et al., 2018). On the other hand, for the sake of driving the application of PHAs, various modified 3D rapid prototyping technologies should be matched with PHA materials. Moreover, physical and chemical modification methods also should be developed to improve their performance for biomedical applications. With the significant alteration of their surface, mechanical properties and regulable rate of degradation, modified PHA polyesters are widely employed for scaffolds to fulfill the requirements of BTE.

**FIGURE 3:**
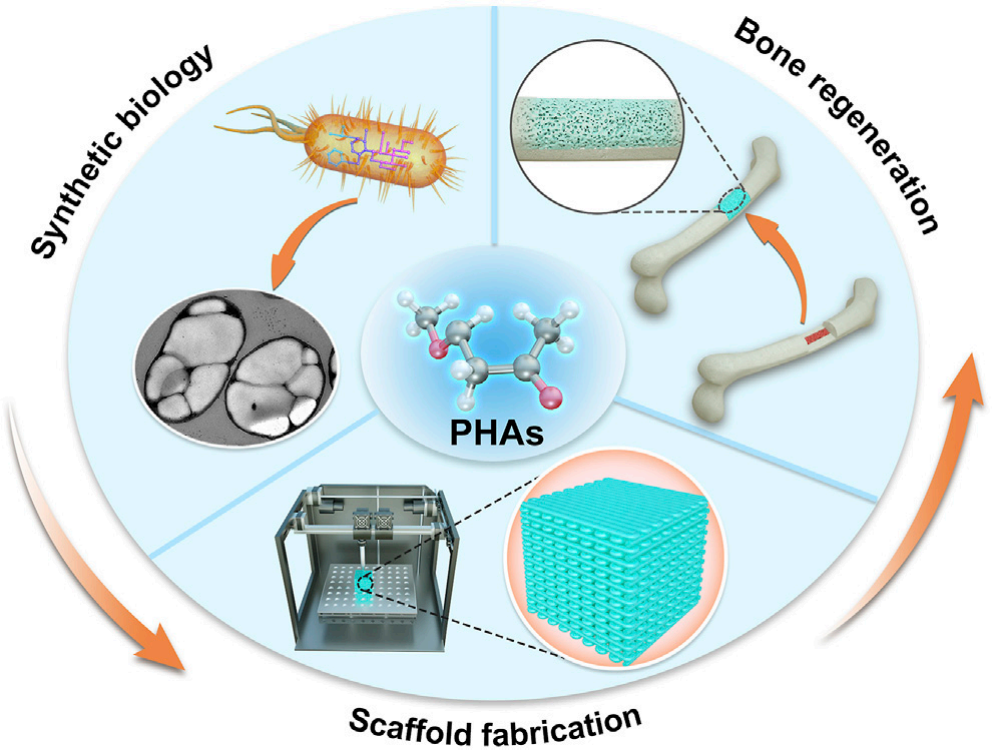
Schematic diagram of the production, fabrication, and applications of PHAs in BTE.

Scaﬀold design requires certain parameters to satisfy the need for tissue regeneration. The recent advances in various methods of PHA scaffolds fabrication were also summarized. Among these scaﬀold fabrication techniques, 3D rapid prototyping has recently been recommended as an advanced method for fabricating diﬀerent microstructures to be suitable for cell growth and rapid nutrient diﬀusion. So far, due to the fewer animal experiments *in vivo* for PHA scaffolds have been investigated, resulting in the limitation of data analysis about immune response of implants to various tissues. Therefore, there is an urge to increase studies on the animal trials of PHAs so that there is more data collected and make them successful biomaterials for bone regeneration. Taken together, developing biomedical applications, especially BTE of PHAs or PHA-based biomaterials not only involves low-cost production, but also needs to consider the fabrication method of PHA scaffolds and the *in vivo* experimental performance before clinical trials (Figure 3).

## Glossary

PHAs: Polyhydroxyalkanoates
PHB: Poly-3-hydroxybutyrate
P4HB: Poly-4-hydroxybutyrate
P3HO: Poly-3-hydroxyoctanoate
P3HP: Poly-3-hydroxyproiponate
scl: Short chain length
mcl: Medium chain length
T_m_: Melting point temperature
T_g_: Glass transition temperature
3HB: 3-hydroxybutyrate
4HB: 4-hydroxybutyrate
3HP: 3-hydroxypropionate
3HV: 3-hydroxyvalerate
3HHx: 3-hydroxyhexanoate
3HO: 3-hydroxyoctanoate
3HD: 3-hydroxydecanoate
3HDD: 3-hydroxydodecanoate
3HTD: 3-hydroxytetradecanoate
scl-*co*-mcl-PHA: (short-*co*-medium)-chain length-PHA
CRISPR: Clustered regularly interspaced short palindromic repeats
RBS: Ribosome-binding site
PhaC: PHAsynthase
PhaA: β-ketothiolase: PhaB: Acetoacetyl-CoA reductase
4HbD: 4-hydroxybutyrate dehydrogenase
SucD: Succinate semialdehyde dehydrogenase
PhaG: 3-hydroxyacyl-ACP:CoA transacylase
PhaJ: enoyl-CoA hydratase
3HB-CoA: 3-hydroxybutyrate-CoA
4HB-CoA: 4-hydroxybutyrate-CoA
3HV-CoA: 3-hydroxyvaleryl-CoA
3HP-CoA: 3-hydroxyproiponate-CoA
3HA-CoA: 3-hydroxyacyl-CoA
P34HB: Poly (3-hydroxybutyrate-*co*-4- hydroxybutyrate)
PHBHHx: Poly (3-hydroxybutyrate-*co*-3-hydroxyhexanoate)
PHBV: Poly (3-hydroxybutyrate-*co*-3-hydroxyvalerate)
P3HHx3HO: Poly(3-hydroxyhexanoate-*co*-3-hydroxyoctanoate)
PHBVHHx: Poly(3-hydroxybutyrate-*co*-3-hydroxyvalerate-*co*-3-hydroxyhexanoate)
OHB: Oligo(3-hydroxybutyrate)
O3HB4HB: Oligo(3-hydroxybutyrate-*co*-4-hydroxybutyrate)
OHBHHx: Oligo(3-hydroxybutyrate-*co*-3-hydroxyhexanoate)
OmclHA: Medium-chain-length oligo(3-hydroxyalkanoate)
PP: Polypropylene
PE: Polyethylene
PEG: Poly(ethylene glycol)
